# Progress on therapeutic mRNA vaccines for cancer

**DOI:** 10.3389/fonc.2026.1980190

**Published:** 2026-10-09

**Authors:** Siyu Wu, Huarong Liao, Wei Zhang

**Affiliations:** 1 Department of Pharmacy, Hubei Provincial Hospital of Traditional Chinese Medicine, Wuhan, China; 2 Hubei Key Laboratory of Theory and Application Research of Liver and Kidney in Traditional Chinese Medicine, Affiliated Hospital of Hubei University of Chinese Medicine, Wuhan, China; 3 Hubei Shizhen Laboratory, Wuhan, China; 4 Hubei Province Academy of Traditional Chinese Medicine, Wuhan, China; 5 Independent Researcher, Wuhan, China

**Keywords:** cancer immunotherapy, combination therapy, innate immune, mRNA vaccine, personalized vaccine, tumor antigen

## Abstract

Therapeutic mRNA vaccines are a clinically validated cancer immunotherapy platform using *in vitro* transcribed mRNA encoding tumor antigens to induce durable anti-tumor immunity. Advances in mRNA engineering, lipid nanoparticle (LNP) delivery, and immunomodulatory combinations have propelled this approach from preclinical models to randomized trials with survival benefit. This review systematically examines vaccine efficacy through an antigen–delivery–immunomodulation tripartite framework. We trace the evolution from shared tumor-associated antigens to personalized neoantigens identified by computational pipelines, discuss ionizable lipid chemistry and LNP formulation for controlled tissue tropism and transfection, and analyze the bidirectional crosstalk between vaccine components and innate immune sensing that shapes adaptive T cell responses. Clinical evidence across melanoma, pancreatic cancer, non-small cell lung cancer, and other solid tumors is critically evaluated, alongside combination strategies—checkpoint blockade, oncolytic virotherapy, and adoptive cell therapy to overcome TME resistance. Persistent challenges include optimal neoantigen selection, standardized immune monitoring, and predictive biomarkers. We conclude that future research should prioritize systematic optimization of the tripartite design through multi-antigen targeting, tissue-specific delivery engineering, and rational combination immunotherapy, aiming to establish mRNA cancer vaccines as a standard-of-care modality.

## Introduction

For over three decades, therapeutic cancer vaccines have been developed as a strategy to activate the adaptive immune system to recognize and attack malignant cells. Therapeutic cancer vaccines must overcome major obstacles, including central and peripheral immune tolerance, tumor-induced immunosuppression, and the genetic and antigenic heterogeneity of advanced cancers, which can differ substantially among patients and within individual tumors (1). Genetic and antigenic heterogeneity is not unique to cancer, as many pathogens also exhibit substantial variability; however, these challenges are particularly difficult in the therapeutic cancer setting. Early attempts using peptide-based vaccines, dendritic cell (DC) vaccines, and viral vectors showed only modest clinical success because of their limited immunogenicity, cumbersome manufacturing, and inability to target patient-specific mutations (2, 3). Three technological breakthroughs have converged to fundamentally change this landscape: the maturation of technologies for mRNA synthesis and modification; the development of ionizable lipid nanoparticle (LNP) delivery platforms capable of efficient *in vivo* transfection of mRNA; and the clinical validation of mRNA vaccines during the COVID-19 pandemic, which established manufacturing infrastructure, regulatory pathways and safety databases at an unprecedented scale (4–6) (**Figure 1**).

**Figure 1:**
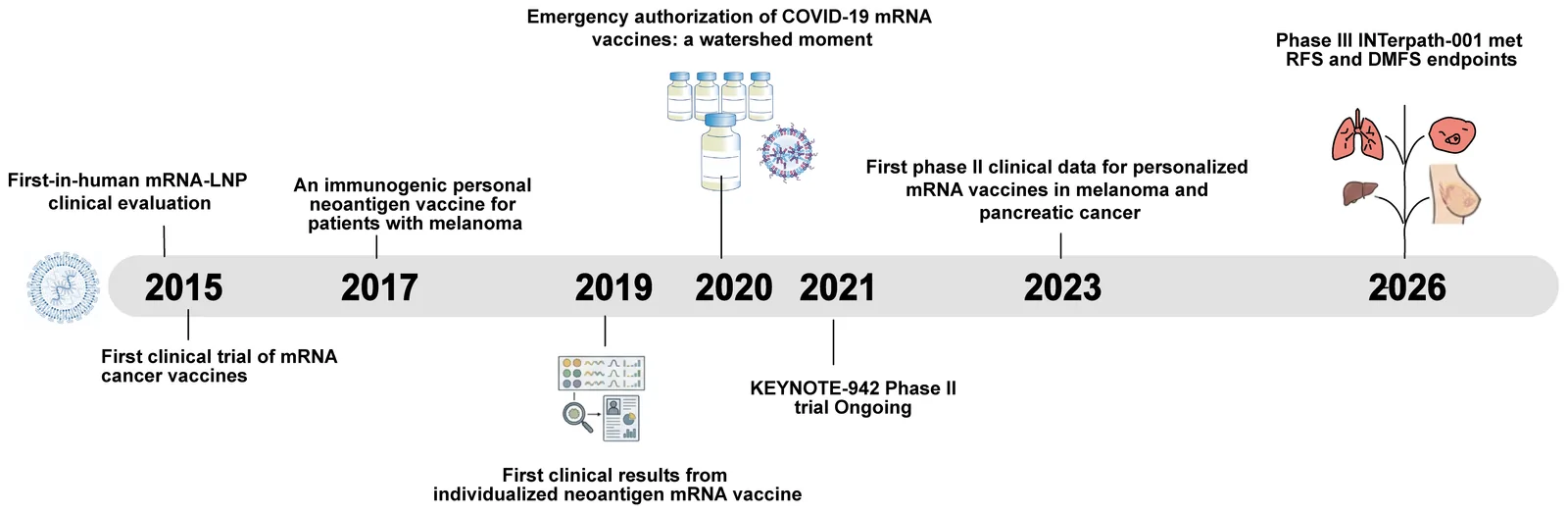
Timeline of major milestones in the development of mRNA cancer vaccines. Key events from 2015 to 2026 are shown, including the initiation of first-in-human clinical trials, the emergence of personalized neoantigen vaccine approaches, and pivotal phase I/II clinical readouts in melanoma and pancreatic cancer. The timeline also highlights the emergency authorization of COVID-19 mRNA vaccines in 2020 as a watershed moment that accelerated the field, followed by ongoing phase II trials (e.g., KEYNOTE-942). The timeline culminates in the phase III INTerpath-001 trial meeting its RFS and DMFS endpoints in 2026. This timeline illustrates the rapid clinical translation of mRNA cancer vaccines from early-stage proof-of-concept to late-stage clinical development over the past decade.

Therapeutic mRNA vaccines are based on a conceptually simple principle. *In vitro* transcribed (IVT) mRNA encoding one or more tumor antigens is delivered to antigen- presenting cells (APCs), mainly dendritic cells, where it is translated into antigenic proteins. These proteins are processed by both MHC class I and class II pathways, priming CD8^+^ cytotoxic T lymphocytes and CD4^+^ T cells that recognize and destroy tumor cells expressing the cognate antigens (7, 8). Unlike DNA vaccines, mRNA does not require nuclear entry or genomic integration, thus avoiding the risk of insertional mutagenesis (9). Unlike peptide vaccines, the mRNA platform encodes the entire antigen, which allows for the presentation of multiple epitopes across a range of different HLA haplotypes. Unlike viral vectors, mRNA is non-infectious and does not have pre-existing anti-vector immunity that can neutralize vaccine efficacy on repeat administration (10). The field of RNA-based therapeutics has rapidly grown with the mRNA vaccines being the most clinically advanced application (11, 12).

In this review, we postulate that the efficacy of therapeutic mRNA cancer vaccines is defined by the interplay of three interdependent design dimensions that we refer to as the antigen-delivery-immunomodulation tripartite framework. The antigen dimension includes the choice of tumor antigen targets, from shared tumor-associated antigens (TAAs) and cancer/testis antigens (CTAs) to personalized neoantigens identified by tumor genome sequencing and computational prediction algorithms (13, 14). The delivery dimension pertains to the engineering of LNP platforms and other delivery vehicles that protect the mRNA cargo, facilitate cellular uptake and endosomal escape and define tissue and cellular tropism (15, 16). The immunomodulation dimension refers to the bidirectional interaction between vaccine components and the host immune system, including the intrinsic adjuvant activity of the mRNA molecules, the extrinsic addition of immunomodulatory agents, and the rational combination with immune checkpoint blockade and other therapies to overcome the immunosuppressive tumor microenvironment (TME) (17, 18). This three-part framework is an analytical lens to compare different vaccine strategies, assess the clinical evidence, and highlight priority directions for future research.

The structure of this review follows the logic of the tripartite framework. We first discuss the antigen dimension, from shared TAA/CTA targets to personalized neoantigen vaccines and mRNA sequence engineering strategies to optimize antigen expression. We then address the delivery dimension, including the ionizable lipid nanoparticle system, structure–activity relationships of its components, formulation optimization, and emerging alternatives. Next, we examine the immunomodulation dimension, following the cascade from innate immune sensing to adaptive T cell priming, TME modulation, and combination immunotherapy. We subsequently provide an overview of the clinical development landscape, manufacturing considerations, and emerging regulatory structures. We then critically review unresolved controversies, evidence gaps, and the strength of the current evidence base across different experimental systems. Finally, we summarize the main findings and outline priority research directions for the next decade.

## Antigen selection and sequence engineering of mRNA cancer vaccines

The choice of the antigen target is the main determinant of the therapeutic specificity in mRNA cancer vaccines. Unlike prophylactic vaccines against pathogens, where conserved structural proteins offer universal targets, cancer vaccines must deal with the extreme antigenic heterogeneity of malignant cells, not only between patients with the same type of cancer but also within individual tumors over time (19). The antigen selection problem can be placed on a continuum of increasing personalization, from common TAAs, which are overexpressed in the tumors of many patients, through CTAs with limited normal tissue expression, to truly personalized neoantigens arising from patient-specific somatic mutations (20). Here, we delineate the stepwise logic of this spectrum, discuss the computational and experimental pipelines that facilitate personalized neoantigen identification, and review the mRNA sequence engineering strategies that work in concert to dictate the magnitude, duration, and immunogenicity of antigen expression (21, 22).

## Tumor-associated antigens and CTAs

Historically, the development of cancer vaccines has been prompted by shared tumor-associated antigens (TAAs) such as melanocyte differentiation antigens (MART-1, gp100, tyrosinase), overexpressed growth factor receptors (HER2, CEA), and oncofetal proteins. These antigens have the practical advantages of off-the-shelf applicability, as a single vaccine construct can treat all patients with tumors expressing the target antigen (23). However, the inherent biological limitation of TAA-targeted vaccines arises from the nature of these antigens, which are self-proteins expressed to some degree in normal tissues, and are subject to the central and peripheral tolerance mechanisms that delete or anergize high-avidity T cells (24). TAA-directed mRNA vaccines typically elicit modest T cell responses with moderate avidity and an intrinsic risk for autoimmune toxicity if the target antigen is expressed in vital normal tissues. Clinical experience with mRNA vaccines encoding TAAs such as CEA in colorectal cancer and HER2 in breast cancer has shown safety and antigen-specific immune responses but objective clinical responses as monotherapy have been limited, suggesting the need for combination with immune checkpoint blockade to overcome tolerance (25).

CTAs like NY-ESO-1, MAGE-A3 and MAGE-A4 occupy a unique immunological niche. These antigens are expressed during gametogenesis, but are epigenetically silenced in adult somatic tissues, but aberrantly reactivated in a broad range of tumor types. As they are only weakly expressed in normal tissues, restricted to immune-privileged testicular germ cells that do not express MHC class I, they are attractive targets with lower risk of autoimmunity than TAAs. mRNA vaccines encoding CTAs have been shown to induce both humoral and cellular immune responses in preclinical models and several constructs have progressed to clinical evaluation. The mRNA-LNP platform encoding CTA has demonstrated the ability to elicit antigen-specific CD8+ T cell responses and suppress tumor growth in mouse models. However, the relatively low frequency of CTA expression in common epithelial cancers, coupled with the heterogeneous intratumoral expression of individual CTAs, limits the patient population that can be benefited by single-CTA vaccines. Multi-CTA mRNA constructs encoding several CTAs at once are a rational approach to broaden the addressable patient population (26).

The TAA/CTA versus personalized neoantigen approaches exemplify the central trade-off in cancer vaccine design of broader patient eligibility at the cost of reduced tumor specificity. TAA- and CTA-based mRNA vaccines are able to be made off-the-shelf for genetically defined subsets of patients, which lowers cost and reduces turnaround time. However, they are inherently limited by immunological tolerance and potential on-target, off-tumor toxicity. These limitations have driven the progressive development of individualized neoantigen vaccines that exploit the unique mutational landscape of each patient’s tumor to drive truly tumor-specific immune responses (27).

## Neoantigen vaccine individually tailored

Neoantigens are peptide epitopes originating from tumor-specific somatic mutations and represent the immunological ideal for cancer vaccine targeting. The absence of the underlying mutations in the normal genome means that the neoantigen-specific T cells are not deleted centrally in the thymus and are not subject to peripheral tolerance mechanisms, which allows the generation of high-avidity effector responses with low risk of autoimmune toxicity (28–30). Synthetic long peptide vaccines first showed the clinical feasibility of neoantigen-targeted vaccines, but the mRNA platform provides unique advantages for this application: rapid manufacturing turnaround (4–6 weeks from tumor sequencing to vaccine administration), the ability to encode multiple neoantigens in a single mRNA construct, and the intrinsic adjuvant activity of mRNA that eliminates the need for separate immunostimulatory agents (31, 32).

The personalized neoantigen vaccine workflow starts with tumor biopsy and matched normal tissue sampling, followed by whole-exome sequencing (WES) and RNA sequencing to identify expressed somatic mutations. Computational pipelines then predict the mutant peptides binding to the patient’s HLA class I and class II molecules at high enough affinity to be displayed on the surface of the tumor cell. Commonly used prediction algorithms include NetMHC and NetMHCpan for MHC class I binding; NetMHCIIpan for MHC class II; and integrated platforms like pVACtools and MuPeXI that include variant calling, HLA typing, peptide processing prediction and prioritization ranking. Critical steps in the filtering process include the selection of clonal over subclonal mutations to minimize the risk of immune escape through antigen loss, the prioritization of mutations in highly expressed genes, and the exclusion of peptides with poor physicochemical properties for MHC binding. This pipeline yields a ranked list of 10–20 mRNA sequences encoding neoantigens, which are then concatenated into a single mRNA construct for vaccine production (33).

A milestone in this area was the demonstration that co-administration of MHC class I- and MHC class II-restricted neoantigens significantly improved the breadth and durability of anti-tumor T cell responses. Cho and colleagues developed an mRNA-based PCV platform encoding both MHC-I and MHC-II neoantigens, and showed that the presence of MHC-II-restricted epitopes was essential to generate robust antigen-specific CD8^+^ T cell responses and durable anti-tumor efficacy in a murine colorectal cancer model (28). Early-stage vaccination induced a more potent anti-tumor effect than late-stage administration, and synergistically enhanced tumor control when combined with immune checkpoint inhibitors.

Clinical evidence supporting the personalized neoantigen approach has been accumulating rapidly. In a key phase I trial of the mRNA neoantigen vaccine autogene cevumeran (BNT122/RO7198457) in patients with resected pancreatic ductal adenocarcinoma, 50% of patients developed *de novo* neoantigen-specific T cell responses, and responders had significantly longer recurrence-free survival than non-responders. In melanoma, the KEYNOTE-942 (mRNA-4157/V940) phase II trial of a personalized mRNA neoantigen vaccine plus pembrolizumab showed a statistically significant improvement in recurrence-free survival compared with pembrolizumab alone, providing the first randomized evidence of clinical benefit of an mRNA cancer vaccine in a controlled setting (34). These results have led to the initiation of phase III trials and highlighted the clinical feasibility of personalized mRNA cancer vaccines (35).

## Optimization of mRNA sequence for increased antigen expression

Intrinsic molecular features of the mRNA molecule itself significantly impact translation efficiency, stability and innate immune recognition (36). A rational approach to sequence engineering has played a key role in transforming mRNA from a labile and poorly translated molecule into a powerful therapeutic platform. Key design parameters are the 5′ cap structure, the 5′ and 3′ untranslated regions (UTRs), the codon composition of the coding sequence, the length of the poly(A) tail, and the nucleoside modification pattern (37).

The most important innovation in mRNA vaccine technology may be the replacement of uridine with N1-methylpseudouridine (m1Ψ). Pardi et al. showed that m1-modified mRNA increases translation efficiency 10- to 100-fold over unmodified mRNA, while decreasing activation of innate immune sensors such as TLR3, TLR7, and TLR8 (2). This dual effect is crucial: enhanced translation increases the amount of antigen produced per mRNA molecule, while reduced innate immune activation prevents the excessive type I interferon responses that can paradoxically suppress translation and promote mRNA degradation (38). The mechanism is based on the altered secondary structure of m1-containing mRNA, which decreases the recognition by RNA-dependent protein kinase (PKR) and 2-5-oligoadenylate synthetase (OAS), thus preventing translational shutdown.

A complementary approach to optimize mRNA expression is UTR engineering. Highly expressed human genes such as α and β globin, heat shock proteins and mitochondrial genes have 5′ and 3′ UTRs that increase mRNA stability and translational output when incorporated into IVT mRNA constructs (39). The 5′ UTR affects ribosome recruitment and scanning efficiency. The 3′ UTR controls mRNA half-life by binding to RNA-binding proteins and microRNAs. Recent advances in high-throughput screening of UTR libraries have allowed identification of synthetic UTR sequences that are superior to natural UTRs in specific cell types.

The advent of circular RNA (circRNA) platforms is an area of mRNA vaccine engineering. Unlike linear mRNA, circRNA has no free 5’ and 3’ ends, thus it is resistant to degradation by exonucleases, leading to prolonged protein expression that can persist for days to weeks (40). The development of efficient *in vitro* circularization methods and the proof-of-concept that circRNA can be formulated in LNPs for *in vivo* delivery have advanced preclinical development of circRNA vaccines. While circRNA-based cancer vaccines are at an earlier stage of development than linear mRNA vaccines, the more prolonged antigen expression kinetics of circRNA may be particularly beneficial for durable T cell memory responses (41).

To summarize, the antigen landscape of mRNA cancer vaccines is marked by the increasing transition from shared TAA/CTA targets to personalized neoantigen approaches (**Table 1**). The comparison of antigen categories emphasizes the inherent trade-off between the broad applicability of shared antigens and the enhanced specificity of personalized neoantigens. Vaccines based on TAA and CTA have the advantage of off-the-shelf manufacturing but are limited by immunological tolerance, while neoantigen vaccines maximize tumor specificity but require individualized manufacturing and longer turnaround times (43). The mRNA sequence optimization strategies discussed in this section, including nucleoside modification, UTR engineering, and circRNA platforms, can benefit all categories of antigen, which together improve the expression, stability, and immunogenicity of the encoded antigens. Computational prediction pipelines for neoantigen identification (**Figure 2**) are an indispensable enabling technology that has transformed personalized cancer vaccination from a concept to a clinical reality (44).

**Table 1:** Targeting mRNA cancer vaccine antigen category comparison.

Antigen category	Tumor specificity	Patient eligibility	Safety profile	Representative targets
TAAs	Moderate; expressed in normal tissues	Broad; off-the-shelf applicability	Risk of autoimmunity	MART-1, gp100, CEA, HER2 (13)
CTAs	High; restricted to testis and tumors	Moderate; expression varies by tumor type	Favorable; immune privileged normal tissue	NY-ESO-1, MAGE-A3, MAGE-A4 (24)
Personalized Neoantigens	Very high; tumor-specific mutations	Individualized; requires sequencing	Excellent; no normal tissue expression	Patient-specific SNV-derived peptides (28)
Viral Oncoproteins	High; viral origin	Restricted to virus-associated cancers	Favorable; non-self-target	HPV E6/E7, EBV LMP2 (42)

**Figure 2:**
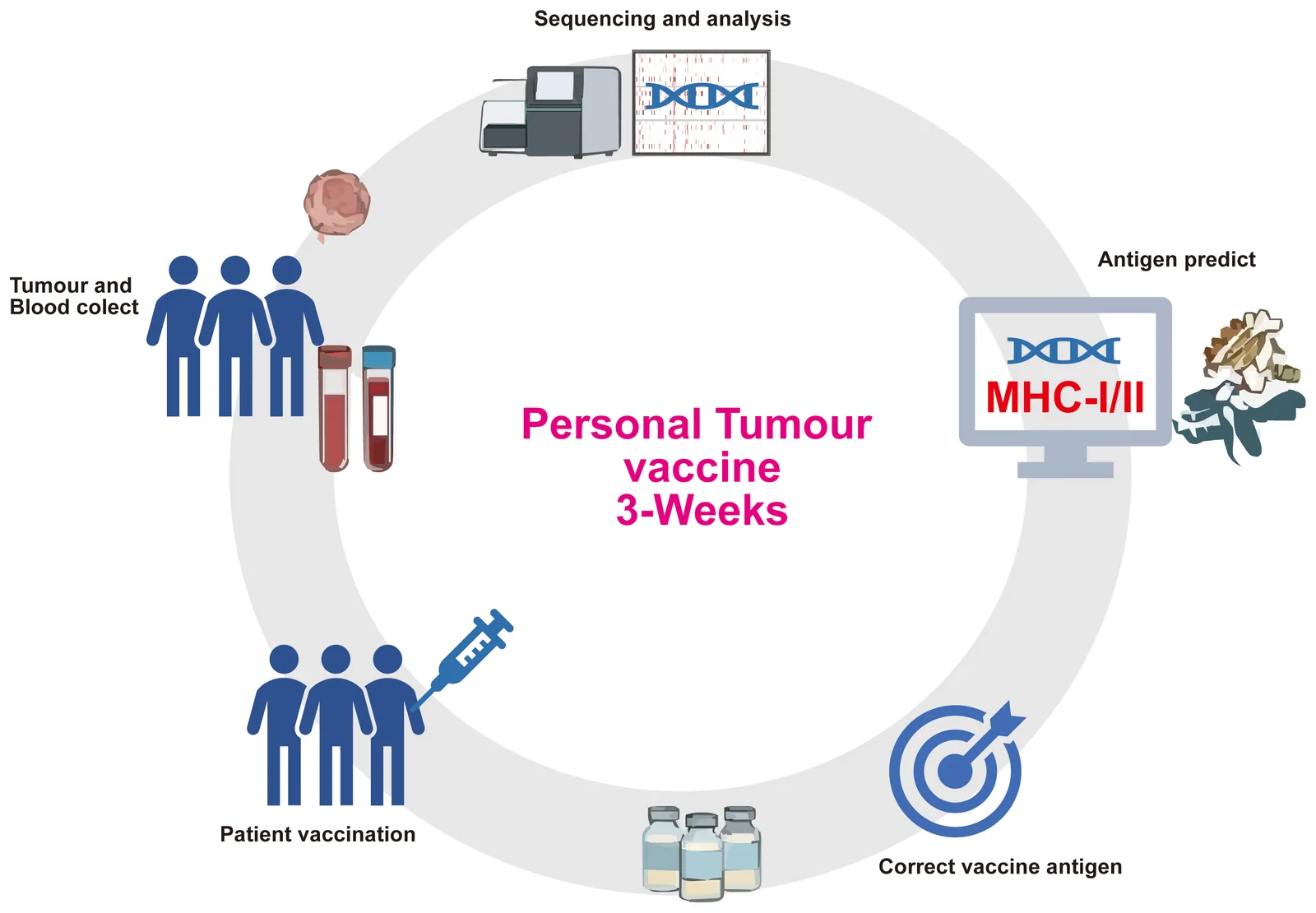
Workflow for the design and manufacture of personalized neoantigen mRNA vaccines. The process begins with tumor tissue and matched blood collection from the patient, followed by whole-exome and RNA sequencing to identify expressed somatic mutations. Computational pipelines then predict MHC class I- and class II-restricted neoantigens based on binding affinity and other prioritization criteria. A personalized mRNA vaccine construct encoding the selected neoantigens is synthesized and manufactured within approximately 3 weeks, and then administered back to the patient. This integrated sequencing–prediction–production pipeline enables rapid turnaround from biopsy to vaccination, which is essential for individualized neoantigen vaccine development.

## Lipid nanoparticle delivery systems for mRNA cancer vaccines

More than any other single factor, it is perhaps the development of ionizable LNP delivery platforms that has enabled the clinical translation of mRNA vaccines. Naked mRNA is inherently unstable in biological fluids, rapidly degraded by extracellular RNases, and cannot cross the anionic cell membrane due to its large size and negative charge (45). The LNP platform is designed to overcome each of these barriers through a four-component architecture that includes an ionizable cationic lipid, a phospholipid, cholesterol, and a PEG-lipid conjugate. The LNP platform binds the mRNA cargo, protects it from degradation and facilitates cellular uptake and endosomal escape (46, 47). Here, we complete the loop of the LNP design cycle, correlating the relationship from ionizable lipid chemistry to nanoparticle formulation optimization and *in vivo* biodistribution to clinical translation (48).

## Ionizable lipid chemistry and structure-activity relationships

The essential functional component of LNPs is the ionizable lipid that is responsible for electrostatic complexation with the anionic mRNA phosphate backbone at acidic pH and for endosomal membrane disruption upon protonation in the acidifying endosomal compartment. The design of ionizable lipids has evolved through several generations: from the early permanently cationic lipids (DOTAP, DOTMA) that exhibited dose-limiting toxicity, to the first clinically validated ionizable lipid DLin-MC3-DMA (used in the FDA-approved siRNA therapeutic patisiran), to the modern lipids SM-102 and ALC-0315 that made the COVID-19 mRNA vaccines possible (49, 50). The structural evolution of ionizable lipids has been defined by three key parameters: the amine headgroup pKa, the linker chemistry between the headgroup and the hydrophobic tails, and the degree of alkyl tail unsaturation (51).

The apparent pKa of the ionizable lipid, which is typically optimized to 6.2–6.5, is a key design parameter that controls the balance between the efficiency of mRNA encapsulation, the circulatory stability, and the potency of endosomal escape. At physiological pH (7.4), the ionizable lipid is predominantly neutral, thus minimizing nonspecific interactions with serum proteins and anionic cell membranes that could lead to rapid clearance or cytotoxicity. Upon endocytosis and endosomal acidification to pH 5.5–6.0, the lipid becomes protonated and cationic, allowing it to electrostatically interact with the negatively charged endosomal membrane lipids and induce membrane disruption that releases mRNA into the cytosol. Ionizable lipids with suboptimal pKa values may show reduced endosomal escape, whereas higher pKa values can increase nonspecific interactions and toxicity at physiological pH.

The linker between the ionizable headgroup and the hydrophobic tails has become an underappreciated determinant of LNP potency and biodistribution. Naidu and colleagues systematically screened a library of ionizable lipids bearing different biodegradable linkers such as ester, amide and urea linkages, and showed that these small structural differences had a profound effect on LNP pKa, biodistribution and *in vivo* mRNA expression (52). Amide and urea linkers modulated the LNP pKa to values that confer improved specificity for lung transfection, and one optimized lipid encapsulating a bacterial toxin mRNA significantly reduced tumor burden and prolonged survival in a murine lung metastasis model. The work emphasizes the necessity of treating the linker as an active design element and not as a passive structural connector (53).

## Alkyl tail architecture—including chain length, degree of unsaturation, and branching pattern—governs the fluidity of the lipid bilayer and the fusogenicity of the LNP

The unsaturated tails introduce kinks in the lipid packing that favor the formation of non-bilayer hexagonal (HII) phases, which are necessary for membrane fusion and endosomal escape (54). However, excessive unsaturation can lead to LNP instability and greater propensity for lipid peroxidation. For SM-102 and ALC-0315, the incorporation of biodegradable ester linkages in the alkyl tails enables rapid metabolic clearance of the ionizable lipid following mRNA delivery. High-throughput combinatorial lipid library screening combined with rational design approaches based on structure-activity relationships has accelerated the discovery of next-generation ionizable lipids with improved therapeutic indices (55).

## Optimization of nanoparticle formulation and biodistribution

Critical factors in the *in vivo* performance of LNPs are their physical properties, such as particle size, polydispersity index, surface PEG density and internal lamellarity, along with the chemical identity of the individual lipid components (47). LNP formulation is commonly performed by rapid microfluidic mixing of an ethanolic lipid solution with an acidic aqueous buffer containing mRNA, which triggers the LNP self-assembly via hydrophobic and electrostatic interactions. Microfluidic mixing parameters such as flow rate ratio, total flow rate, and lipid to mRNA ratio (N/P ratio) control the nucleation and growth kinetics that determine the final size distribution and encapsulation efficiency of LNPs. Optimized formulations generally produce LNPs of 60–100 nm in diameter, with encapsulation efficiencies >90% and low polydispersity (<0.2) (56).

The PEG-lipid component, usually present at 1.5–2.5 mol% of total lipid, plays a critical dual role in LNP performance. PEG-lipids stabilize the surface of the nascent LNP and prevent aggregation of particles during formulation. The PEG corona in circulation forms a steric barrier that reduces opsonization and clearance by the mononuclear phagocyte system, thereby extending the circulation half-life (57). However, the trade-off with PEG is well known: PEG-lipids with long acyl chains dissociate slowly from the LNP surface, stabilizing the formulation but reducing cellular uptake, while PEG-lipids that dissociate rapidly improve transfection but reduce colloidal stability. The design of PEG-lipids with optimized dissociation kinetics to address these competing requirements is an active area of research (58).

A hallmark of conventional LNPs is their predominant liver tropism after intravenous administration. This is because apolipoprotein E (ApoE) gets adsorbed onto the LNP surface in circulation, which leads to receptor-mediated uptake by hepatocytes expressing the low-density lipoprotein receptor (LDLR) (59). Liver tropism has been beneficial for hepatic indications and for hepatic expression of secreted proteins, but it is a major limitation for cancer vaccine applications where delivery to dendritic cells in lymphoid organs or directly to the tumor is desirable. To divert LNP biodistribution away from the liver, strategies include incorporating negatively charged lipids that limit ApoE adsorption, using alternative routes of administration (intramuscular, intradermal, intratumoral), and engineering active targeting ligands on the LNP surface (60–62).

Intramuscular injection, as used for the COVID-19 mRNA vaccines, leads to local transfection of muscle cells, fibroblasts and resident immune cells at the injection site and some LNPs draining to regional lymph nodes. This route has been demonstrated to effectively induce strong humoral and cellular immune responses, with the local inflammatory response induced by the injection acting as a natural adjuvant that promotes dendritic cell recruitment and maturation (63). Examples of lymph node-targeting strategies that have shown potential to increase vaccine potency by direct delivery of mRNA to the anatomical location of T cell priming include engineered LNPs with optimal size (<50 nm) for passive lymphatic drainage and the addition of albumin-binding lipids to improve lymphatic transport. Luo et al. (64) designed a spleen-targeted mRNA vaccine with manganese adjuvant to enhance the STING pathway and strong anti-cancer immunity *in vivo*, showing the potential of targeted delivery to secondary lymphoid organs (65, 66).

## New technologies and alternative platforms for delivery

However, LNPs are the clinical standard today, and a broad range of alternative delivery platforms provide distinct advantages for specific applications (67). Cationic polymer-based systems such as polyethyleneimine (PEI), poly (β-amino esters) (PBAEs) and dendrimers can efficiently complex with mRNA via electrostatic interactions and have been engineered to contain pH-responsive or biodegradable properties to improve the safety profile (68). Polymer-based systems offer a higher degree of chemical diversity in fine tuning charge density, molecular weight and degradation kinetics than LNPs. However, their clinical translation for cancer vaccine applications has been limited by the inherent polydispersity of polymer synthesis, the potential for complement activation upon intravenous administration, and the generally lower transfection efficiency compared with optimized LNPs.

Biomimetic delivery systems are a conceptually different approach that exploits nature’s own solutions for cellular communication (69). Synthetic nanoparticle cores cloaked with membranes from cancer cells, immune cells or red blood cells (so-called cell membrane-coated nanoparticles) integrate the targeting and immune-evasive properties of biological membranes with the cargo-loading capacity of synthetic carriers. Sun et al. engineered an ultrasound-responsive nanovaccine that was loaded with engineered cancer cell membrane and RNA and showed the ability to prevent metastasis in a murine model (70). Virus-like particles (VLPs) and engineered viral vectors based on arenaviruses or alphaviruses have the advantage of natural tropism for APCs and the capacity to deliver self-amplifying mRNA, but are limited by manufacturing complexity and pre-existing anti-vector immunity (71).

Notably, emerging technologies include carbon dot-lipidosis nanocarriers for mucosal vaccination (72), injectable hydrogel scaffolds for sustained local release of mRNA vaccines at the tumor site, and implantable devices for *in situ* production of personalized mRNA vaccines. These approaches are still in the early stages of development and address specific limitations of bolus LNP administration, such as the short duration of antigen expression and the need for repeated dosing (73). The comparative assessment of LNPs and these alternative platforms, in terms of potency, manufacturability, safety and clarity of regulatory pathway, suggests that LNPs will likely remain the dominant platform for clinical work in the foreseeable future, whereas alternative platforms may find niche applications where their specific advantages are most impactful (74).

The LNP delivery landscape presented in **Table 2** and illustrated in **Figure 3** shows a clear trend toward lipids with improved biodegradability and tissue-specific tropism. Comparison of clinical and preclinical ionizable lipid performance reveals the trade-off between transfection efficiency and tissue selectivity. The lipids of COVID-19 mRNA vaccines, SM-102 and ALC-0315, exhibit high transfection efficiency due to their ester-based biodegradability, but, their liver tropism hinders their application in cancer vaccines requiring extrahepatic delivery (76). The development of lung-specific ionizable lipids with amide or urea linkers as demonstrated by Naidu and colleagues exemplifies a paradigm shift from the one size fits all LNP to tissue-specific targeted formulations which can be matched to the anatomical requirements of specific cancer indications. The closed loop relationship between lipid chemistry, formulation parameters and biodistribution will continue to drive the evolution of LNP technology toward the next generation of cancer vaccines (77).

**Table 2:** Comparative performance of ionizable lipids used in preclinical and clinical mRNA cancer vaccines.

Ionizable lipid	pKa	Transfection efficiency	Biodegradability	Liver tropism	Reference
DLin-MC3-DMA	6.44	Moderate	Low	High	(75)
SM-102	6.68	High	High (ester)	High	(49)
ALC-0315	6.09	High	High (ester)	High	(49)
Lipid 35 (amide)	6.2–6.5	Moderate	High (amide)	Low (lung-specific)	(52)
LP-01 (proprietary)	6.0–7.0	High	High	High	(54)

**Figure 3:**
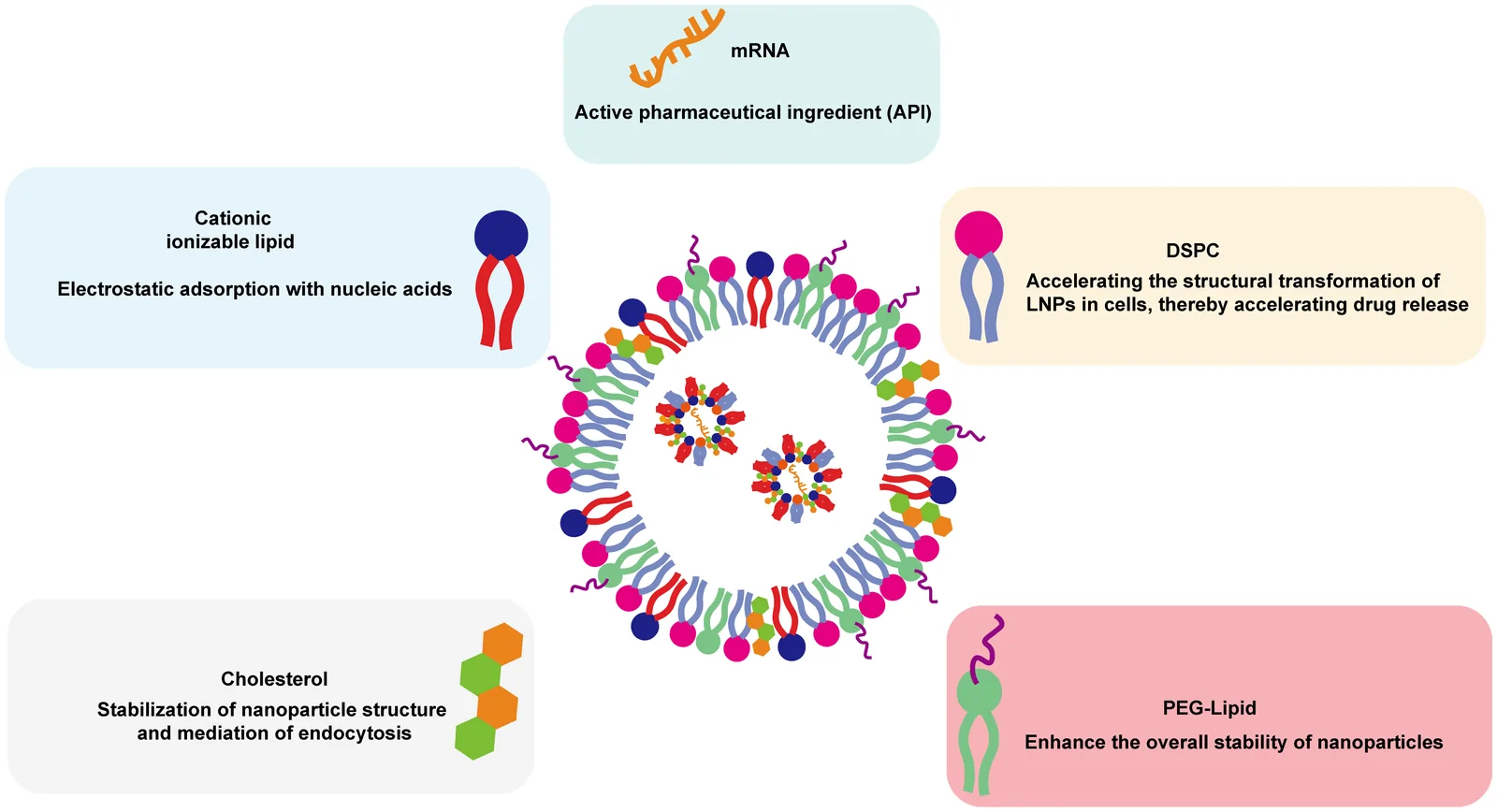
Architecture of ionizable lipid nanoparticles (LNPs) for mRNA delivery. LNPs are typically composed of four lipid components: an ionizable cationic lipid, designed to electrostatically complex with the negatively charged mRNA payload and facilitate endosomal escape upon protonation; DSPC (1,2-distearoyl-sn-glycero-3-phosphocholine), a phospholipid that stabilizes the nanoparticle structure and mediates cellular uptake; cholesterol, which enhances overall particle stability and modulates membrane fluidity; and PEG-lipid conjugates, which form a surface corona to prevent aggregation and extend circulation half-life. The mRNA encoding the tumor antigen is encapsulated in the aqueous core as the active pharmaceutical ingredient (API). This multicomponent design is critical for protecting the mRNA cargo, enabling efficient transfection, and achieving potent antigen expression *in vivo*.

## Immunomodulatory mechanisms and combination therapies

The efficacy of mRNA cancer vaccines depends not only on the antigen encoded and the efficiency of delivery but also on the nature and magnitude of the immune response induced. Unlike conventional vaccines that require exogenous adjuvants to generate the “danger signals” necessary to elicit an immune response, mRNA vaccines are in a unique immunological position: the mRNA molecule itself is immunostimulatory and activates several pattern recognition receptor (PRR) pathways, thereby serving as an intrinsic adjuvant. This section discusses the stepwise cascade from innate immune sensing of mRNA to adaptive T cell priming and memory formation, the complex interplay with the immunosuppressive TME, and the evidence base for combination strategies that target immune evasion mechanisms at each stage.

## Innate immune sensing of mRNA vaccines

The innate immune system detects exogenous mRNA via two major classes of PRRs: the endosomal Toll-like receptors TLR3 (dsRNA), TLR7 and TLR8 (ssRNA), and the cytosolic RIG-I-like receptors (RLRs) RIG-I and MDA5 (dsRNA with 5’-triphosphate or uncapped structures). These sensors upon activation induce signaling cascades, mainly via the MyD88/TRIF adaptors for TLRs and the MAVS adaptor for RLRs, converging on the transcription factors NF-κB and IRF3/IRF7, resulting in the production of type I interferons and pro-inflammatory cytokines. This innate activation is the key “signal” that licenses dendritic cells for antigen presentation, increasing MHC molecules, co-stimulatory ligands (CD80/CD86) and chemokine receptors that guide them to T-cell zones of secondary lymphoid organs.

Paradoxically, the relationship between innate sensing and vaccine efficacy is not monotonic. Overactivation of type I interferon responses, particularly those initiated by the MDA5-MAVS pathway, can inhibit mRNA translation through activation of PKR and OAS, promote mRNA degradation through recruitment of RNase L, and induce dendritic cell apoptosis, resulting in reduced antigen availability for T cell priming. This m1 modification is a rational way to mitigate this excessive innate activation: m1-modified mRNA reduces the recognition by TLR3, TLR7, and TLR8, resulting in a fine balance where a sufficient level of innate activation is maintained to induce DC maturation without triggering the translational shut-off limiting the expression of the antigen. Recently, Loomis and colleagues demonstrated that IVT mRNA vaccines with programmable stimulation of innate immunity could be engineered by modulating the nucleoside modification pattern, providing a proof-of-concept for rational tuning of innate immune activation (38).

The type I interferon response also has an important temporal dimension. Early transient IFN-α/β production promotes DC cross-presentation and CD8+ T cell priming, while sustained or excessive IFN signaling may suppress T cell responses through the induction of PD-L1, IL-10 and indoleamine 2,3-dioxygenase (IDO) in the TME. This window of opportunity where innate activation enhances rather than inhibits adaptive immunity is governed by the kinetics of mRNA translation, the rate of LNP-mediated delivery of mRNA to DCs and the cytokine induction profile which is dependent on formulation. The rational design of LNPs with tunable mRNA release kinetics, combined with judicious use of nucleoside modifications, provides a promising strategy to optimize the temporal profile of innate activation for maximal vaccine efficacy (78, 79).

## Adaptive T cell responses and immune memory

The ultimate therapeutic goal of mRNA cancer vaccines is the induction of robust, durable and tumor-specific T cell responses. The antigen presentation pathway bifurcates into two complementary routes: direct presentation, in which LNPs transfect DCs that then translate the mRNA and present the encoded antigens on their own MHC molecules, and cross-presentation, in which non-immune cells at the injection site produce the antigen protein that is later taken up and processed by DCs. Both pathways contribute to the overall T cell response, but the relative contribution of each pathway is dependent on the tropism of the LNP formulation, the route of administration and the nature of the encoded antigen. In fact, there is evidence that lymph node-resident macrophages are important for mediating innate immune responses that augment the anti-tumor efficacy of mRNA vaccines, suggesting that the cellular target of LNP delivery is as important as transfection efficiency (80).

The quality of the T cell response, as defined by the magnitude, polyfunctionality (the simultaneous production of multiple effector cytokines including IFN-γ, TNF-α and IL-2) and memory differentiation, is a more reliable predictor of anti-tumor efficacy than magnitude alone (81). Clinical studies of mRNA vaccines have consistently demonstrated induction of both CD8^+^ and CD4^+^ T cell responses, with polyfunctional T cell phenotypes associated with clinical benefit. The inclusion of MHC class II-restricted epitopes in mRNA vaccine constructs has been identified as critical for the generation of CD8^+^ T cell memory because CD4^+^ T cell help is necessary for the programming of memory precursor effector cells and the maintenance of memory T cell populations. The landmark clinical results of the personalized neoantigen vaccine trials, in which T cell responses persisted months to years after vaccination and were associated with prolonged recurrence-free survival, provide clinical validation of the importance of durable memory T cell responses for sustained tumor control.

Increasingly, the mechanisms of immune evasion that limit vaccine efficacy in the clinical setting are becoming well understood. Tumor cells can escape CD8^+^ T cell attack by downregulating MHC class I expression, making them invisible to CD8^+^ T cells, upregulating inhibitory ligands such as PD-L1 that bind to PD-1 on activated T cells and induce T cell exhaustion, and recruiting immunosuppressive cell populations such as regulatory T cells (Tregs), myeloid-derived suppressor cells (MDSCs) and tumor-associated macrophages (TAMs) that actively suppress effector T cell function (82, 83). These mechanisms account for the fact that the mere induction of tumor-specific T cells by vaccination is often not sufficient for clinical efficacy and that combination strategies targeting immune evasion mechanisms are necessary to unleash the full therapeutic potential of mRNA cancer vaccines (84, 85).

## Combination immunotherapy and TME modulation

The immunosuppressive TME is the major barrier to clinical efficacy of mRNA cancer vaccines. The notion of cold versus hot tumors, distinguished by the presence or absence of T cell infiltration and an inflammatory gene signature, has arisen as a clinically useful framework to comprehend the differential responsiveness of different tumor types to immunotherapy (86). mRNA vaccines are capable of converting cold tumors into hot tumors, by inducing *de novo* T cell responses, and providing the initial T cell priming that is absent in poorly immunogenic tumors (87). However, the TME actively resists this conversion by several complementary mechanisms that need to be overcome by rational combination therapy.

The most clinically advanced approach to overcome TME-driven immunosuppression is based on the combination of mRNA vaccines with immune checkpoint inhibitors (ICIs) targeting the PD-1/PD-L1 and CTLA-4 axes. The mechanistic rationale is compelling: mRNA vaccination induces tumor-specific T cells that infiltrate the tumor and produce IFN-γ, which upregulates PD-L1 expression on tumor cells and myeloid cells as an adaptive resistance mechanism that can be blocked with anti-PD-1/PD-L1 antibodies (88). The phase II KEYNOTE-942 trial of mRNA-4157 in combination with pembrolizumab in high-risk melanoma provided the first randomized evidence that this combination can improve recurrence-free survival compared with pembrolizumab alone, validating the clinical relevance of the combination approach. Clinical evidence is also emerging for the combination of mRNA vaccines with anti-CTLA-4 antibodies, which promote T cell priming by blocking the CTLA-4-mediated suppression of CD28 co-stimulation at the DC–T cell interface.

Beyond checkpoint blockade, a number of combination strategies are under investigation. The combination of mRNA vaccines and oncolytic viruses (OVs) exploits the ability of OVs to induce immunogenic tumor cell death, release of tumor antigen and damage-associated molecular patterns (DAMPs), and remodeling of the TME toward an inflammatory TME permissive to T cell infiltration (89, 90). Combination therapy with an OV and an mRNA vaccine encoding tumor-associated antigens significantly reduced tumor burden and prolonged survival in a murine melanoma model. Combination therapy mediated superior cytokine and chemokine induction within tumors and increased CD8^+^ T cell recruitment as compared to either modality alone. The heterologous prime-boost approach, in which an OV or other viral vector primes the initial T cell response and an mRNA vaccine boosts it, is especially promising for overcoming intratumoral immune suppression.

Combination of mRNA vaccines with adoptive cell therapy (ACT), including CAR-T cells and tumor-infiltrating lymphocytes (TILs), is an emerging strategy overcoming complementary limitations of each modality. While ACT can provide a bolus of tumor-reactive T cells with the ability to kill tumors immediately, the immunosuppressive TME often limits the persistence and functionality of transferred T cells. mRNA vaccination can be a continuous source of antigen for T cell stimulation and has the potential to enhance expansion, persistence and effector function of adoptively transferred T cells. The combination of mRNA vaccines with STING agonists, TLR agonists and other innate immune activators is another level of immunomodulation that is being actively investigated in preclinical models and early-phase clinical trials (91).

The immunomodulatory cascade (**Figure 4**) and TME challenge-strategy matrix (**Table 3**) together describe the current landscape of combination immunotherapy for mRNA cancer vaccines. The key insight is that no single immunomodulatory strategy can overcome all resistance mechanisms utilized by tumors. The optimal therapeutic regimens will probably combine mRNA vaccines with complementary modalities that target different immune evasion mechanisms, including checkpoint blockade to reverse T cell exhaustion, oncolytic viruses to enhance T cell infiltration and STING agonists to amplify innate immune activation (93). The challenge for the field is to identify the optimal combination for each tumor type and clinical setting, using predictive biomarkers of response and resistance as a guide.

**Figure 4:**
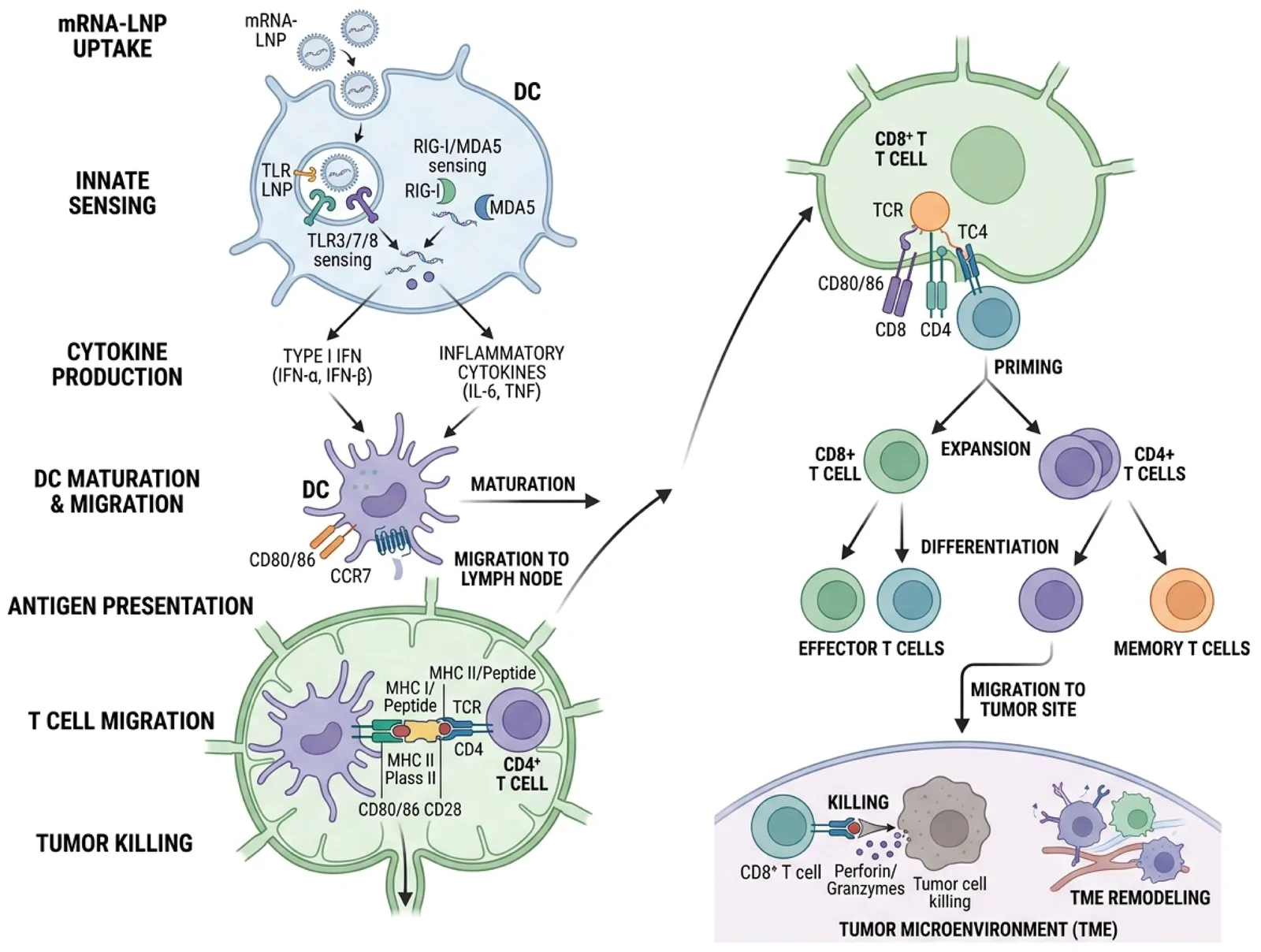
From innate immune sensing of mRNA-LNP vaccines to adaptive anti-tumor T cell responses. Following administration, mRNA-LNP vaccines are taken up by antigen-presenting cells (APCs) such as dendritic cells (DCs). The mRNA cargo is recognized by endosomal Toll-like receptors (TLR3, TLR7, and TLR8), triggering innate immune signaling cascades that lead to type I interferon (IFN-α/β) and pro-inflammatory cytokine production. This innate activation promotes DC maturation, characterized by upregulation of costimulatory molecules (CD80/CD86) and MHC class I/II molecules, and facilitates DC migration to secondary lymphoid organs. There, DCs present antigenic peptides via MHC-I to CD8^+^ T cells and via MHC-II to CD4+ T cells, priming antigen-specific T cell responses. Primed CD8+ cytotoxic T lymphocytes migrate to the tumor site and eliminate tumor cells through perforin/granzyme-mediated cytotoxicity, while CD4^+^ helper T cells provide critical support for sustaining CD8^+^ T cell function and memory formation. This integrated cascade—from innate sensing to DC maturation, antigen presentation, and T cell effector function—underpins the therapeutic efficacy of mRNA cancer vaccines.

**Table 3:** TME challenges and relevant combination strategies for mRNA cancer vaccines.

Challenge	Manifestation	Current strategies	Future directions
T cell exhaustion	PD-L1 upregulation; TIM-3, LAG-3 expression	Anti-PD-1/PD-L1 antibodies (83)	Multi-checkpoint blockade; bispecific antibodies
Immune-cold TME	Lack of T cell infiltration; low mutational burden	Oncolytic viruses (89); STING agonists	*In situ* vaccination; epigenetic reprogramming
Treg and MDSC infiltration	TGF-*β*, IL-10 secretion; arginase-1 expression	Anti-CTLA-4 (83); low-dose cyclophosphamide	Treg-specific depletion; MDSC differentiation therapy
Antigen loss	MHC-I downregulation; *β*2-microglobulin mutation	Multi-antigen targeting (28)	Epigenetic MHC-I upregulation; NK cell engagement
Immune memory failure	T cell exhaustion; poor memory differentiation	MHC-II epitope inclusion (28)	circRNA for prolonged antigen expression (92)

## Clinical development, manufacturing and regulatory setting

Promising preclinical platforms of mRNA cancer vaccines need to be translated into registered therapeutic products with integrated advances in clinical development strategy, manufacturing process engineering and regulatory framework adaptation (94). The COVID-19 mRNA vaccine experience has been an unprecedented accelerator for all three dimensions, building safety databases in the billions of doses, demonstrating the scalability of GMP-compliant mRNA-LNP manufacturing, and setting regulatory precedent for accelerated approval of mRNA-based products (95). This section reviews the current clinical trial landscape, discusses the manufacturing process from the plasmid DNA template to sterile fill-finish, and reviews the evolving regulatory structures governing the development of personalized cancer vaccines (96).

## Clinical trial landscape by tumor type

Melanoma has been the most advanced clinical indication for mRNA cancer vaccines, reflecting the well-established immunogenicity of this tumor type and the precedent of CTLA-4 and PD-1 blockade as standard-of-care therapies (97). In the phase IIb KEYNOTE-942 (mRNA-4157/V940) trial, patients with high-risk resected stage IIIB–IV melanoma were randomized to receive a personalized mRNA neoantigen vaccine encoding up to 34 neoantigens in combination with pembrolizumab versus pembrolizumab alone. The trial met its primary endpoint of recurrence-free survival, with the combination arm showing a statistically significant improvement over the control arm. These results, the first randomized evidence of clinical benefit for an mRNA cancer vaccine in a controlled setting, have led to the initiation of the phase III INTerpath-001 trial and Breakthrough therapy designation from the FDA.

Among these, the results of the phase I study of autogene cevumeran (BNT122) in pancreatic ductal adenocarcinoma (PDAC), a tumor type with a low mutational burden, dense desmoplastic stroma, and resistance to checkpoint blockade, were particularly notable. Rojas et al. showed that 50% of resected PDAC patients developed *de novo* neoantigen-specific T cell responses following vaccination and that responders had significantly prolonged recurrence-free survival (median not reached at 18 months vs 13.4 months in non-responders). The association of vaccine-induced T cell responses with clinical outcome, and the demonstration that neoantigen-specific T cells can persist for years and traffic to the site of the tumor, provide a mechanistic rationale for the clinical benefit and support the further development of personalized mRNA vaccines in immunologically challenging tumor types.

Clinical development has also focused on non-small cell lung cancer (NSCLC), with several mRNA vaccine candidates evaluated in phase I and II trials, including mRNA-5671 targeting KRAS mutations and BNT116 targeting tumor-associated antigens for NSCLC (98). The combination of KRAS G12V mRNA vaccine and pembrolizumab has shown clinical benefit in patients with advanced solid tumors, with evidence of KRAS-specific T cell responses and tumor regression (96, 99). The early clinical experience with mRNA vaccines in colorectal cancer and gastrointestinal tumors has been more limited but promising, with preclinical evidence supporting the feasibility of mRNA neoantigen vaccines in microsatellite stable (MSS) tumors (100). Emerging clinical data across tumor types show a consistent pattern in which the magnitude of vaccine-induced T cell responses correlates with clinical benefit, and the combination of mRNA vaccines with checkpoint blockade consistently outperforms either modality alone (101) (**Figure 5**).

**Figure 5:**
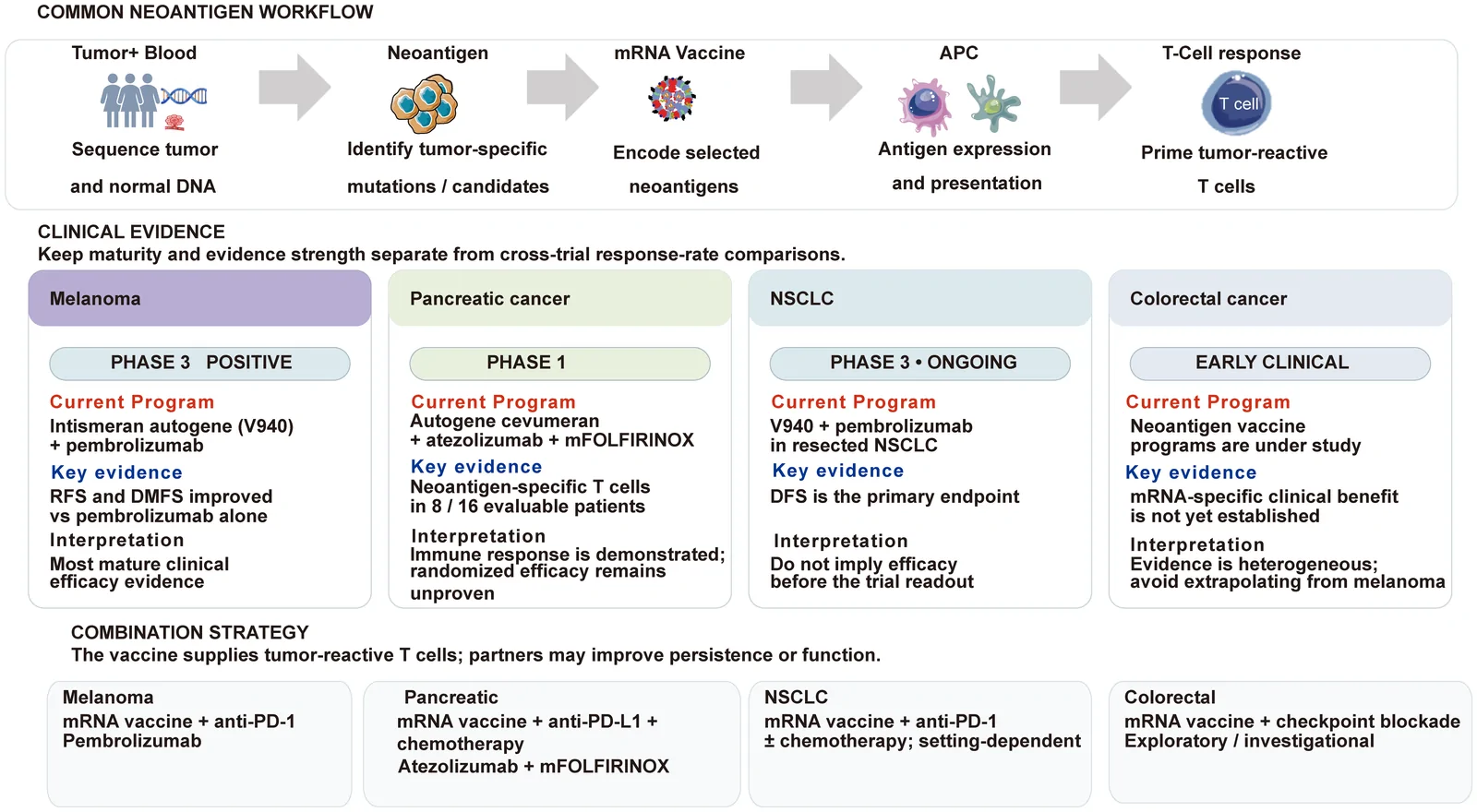
Clinical landscape of personalized mRNA neoantigen vaccines across major cancer types. Clinical development is most advanced in melanoma, where Phase 3 evaluation of intismeran autogene (V940; mRNA-4157) plus pembrolizumab has demonstrated improvements in recurrence-free and distant metastasis-free survival. In pancreatic cancer, Phase 1 studies of autogene cevumeran have demonstrated neoantigen-specific T-cell immunogenicity, with vaccine-induced responses associated with delayed recurrence. V940-based therapy remains under Phase 3 evaluation in resected NSCLC, whereas mRNA-specific clinical benefit in colorectal cancer remains unestablished. Combination strategies primarily include immune checkpoint blockade, with chemotherapy incorporated in selected clinical settings.

mRNA vaccines are being investigated in pediatric solid tumors (102), prostate cancer (103), and hepatocellular carcinoma, in addition to the major solid tumor indications. Experience with mRNA vaccines during the COVID-19 pandemic has also informed the clinical development of cancer vaccines, particularly for safety monitoring and management of rare adverse events (104).

## GMP manufacturing and scale-up

The production of mRNA vaccines is a multi-step process with a sequence of unit operations that must be performed under GMP conditions and strict quality control at each step. The process starts with the production of a plasmid DNA (pDNA) template in Escherichia coli coding for the antigen sequence under the control of a T7, T3 or SP6 bacteriophage promoter. The pDNA is digested and purified with restriction enzymes. This pDNA is used as a template for *in vitro* transcription (IVT) with T7 RNA polymerase, producing the mRNA transcript with the desired nucleoside modifications (typically m1 instead of uridine) and cap analog (typically CleanCap or ARCA). After the IVT reaction, the template DNA is degraded by DNase treatment, the mRNA is purified by chromatography or precipitation to remove double-stranded RNA (dsRNA) contaminants that can induce excessive innate immune activation and the product is formulated in the appropriate storage buffer.

The LNP formulation step is generally performed by microfluidic mixing of the ethanolic lipid solution with the acidic aqueous mRNA solution, which results in the self-assembly of LNPs with controlled size and encapsulation efficiency. The prepared LNPs were buffer-exchanged by tangential flow filtration (TFF) to remove ethanol and adjust pH to physiological conditions, sterile-filtered, and filled into vials. One of the major advantages of the mRNA platform compared to peptide- or DC-based approaches that have much longer timelines for manufacturing is the ability to complete the entire process from pDNA template to the final drug product in as little as 4–6 weeks for a personalized vaccine.

The COVID-19 vaccine experience has convincingly shown the scalability of mRNA manufacturing, with billions of doses produced in the first two years of the pandemic (105). The platform nature of mRNA manufacturing, where the same process, equipment and facilities can be used to make different mRNA sequences by changing the pDNA template, is particularly suited to personalized cancer vaccines, where each patient is treated with a different construct. A key role in facilitating the rapid clinical translation of personalized mRNA vaccines has been played by the concept of “platform validation”, where the manufacturing process, analytical methods and quality control systems are validated independent of the specific mRNA sequence. There are still many hurdles to overcome, including cold-chain distribution requirements (generally C to C for LNP-formulated mRNA), the cost and complexity of individualized manufacturing at scale and the need for rapid, robust potency assays that can be performed within the short turnaround time required for customized vaccines (81).

## Regulatory routes and quality norms

The regulatory landscape for mRNA cancer vaccines remains in flux, influenced by the precedent of COVID-19 vaccine authorizations and the distinct challenges of personalized products (106). The biggest regulatory challenge is to come up with quality standards that can accommodate the inherent variability of personalized vaccines, where every patient receives a different mRNA sequence, but that also ensure consistent safety and potency. The FDA and EMA have issued guidance on the development of personalized mRNA cancer vaccines acknowledging the need for a risk-based approach that focuses on the consistency of the manufacturing process and the characterization of the platform rather than the extensive characterization of each individual vaccine lot.

Regulatory pathways for mRNA cancer vaccines include accelerated approval based on surrogate endpoints (e.g., recurrence-free survival or pathological complete response), breakthrough therapy designation for products that demonstrate substantial improvement over available therapy, and conditional marketing authorization in the EU. The known safety profile of the mRNA-LNP platform and the clinical validation of mRNA cancer vaccines in randomized phase II and III trials have strengthened the regulatory case for these products. However, demonstration of long-term clinical benefit in registrational trials, standardization of immune monitoring assays for neoantigen-specific T cell responses and development of potency assays correlating with clinical efficacy remain important regulatory priorities (107). .

In the phase IIb trial of mRNA-4157/V940 plus pembrolizumab in 157 patients with high-risk melanoma, recurrence-free survival was significantly improved versus pembrolizumab alone, offering the first randomized evidence of clinical benefit for an mRNA cancer vaccine. In pancreatic cancer, a phase I trial of autogene cevumeran (BNT122) in 16 patients with resected PDAC demonstrated that 50% of patients developed *de novo* neoantigen-specific T cell responses and responders had significantly longer recurrence-free survival. The combination of a KRAS G12V mRNA vaccine with pembrolizumab has shown clinical benefit with evidence of KRAS-specific T cell responses in patients with advanced solid tumors, whereas BNT111 (FixVac) in combination with cemiplimab demonstrated improved objective response rates in 184 patients with anti-PD-1-refractory melanoma. Taken together, the randomized phase II data in melanoma, the phase I data in pancreatic cancer, and the emerging evidence in NSCLC provide clinical proof of concept for mRNA cancer vaccines across multiple tumor types and clinical settings. The simultaneous evolution of GMP manufacturing scalability and regulatory framework adaptation has set the stage for the transition of mRNA cancer vaccines from investigational agents to registered therapeutic products (108).

## Research gaps, controversies, and strength of evidence

Significant progress has been achieved in the development of mRNA cancer vaccines, but many important questions and evidence gaps persist. The field has progressed rapidly on the basis of clinical data, but a number of fundamental questions remain incompletely answered, and the strength of the evidence base varies substantially across domains. Here we critically discuss the major controversies, evaluate the strength of evidence from different experimental systems, and identify the gaps that need to be filled to bring the field to its full clinical potential.

One of the most important open questions is the optimal number and criteria for selection of neoantigens in personalized vaccines. Current clinical protocols typically use 10–34 neoantigens per vaccine, selected based on predicted MHC binding affinity, gene expression level and clonality. However, there is no consensus on the optimal number of neoantigens and the relative importance of these selection criteria has not been prospectively validated in clinical trials. Increasing the number of neoantigens may broaden the T cell response and reduce the risk of immune escape by loss of antigen, but it also the greater the complexity of manufacturing and the risk of incorporating subdominant epitopes which may compete with immunodominant epitopes for T cell priming. The development of functional assays that can directly measure the immunogenicity of predicted neoantigens, rather than relying on computational prediction alone, would allow for more rational neoantigen selection and could improve vaccine efficacy (33).

The relative importance of CD4^+^ versus CD8^+^ T cell responses for clinical efficacy is an active topic of debate. There is a good reason to focus on the CD8^+^ T cell response. CD8^+^ cytotoxic T lymphocytes are the main effector cells that directly kill tumor cells. However, clinical evidence is increasingly demonstrating that CD4^+^ T cell responses can provide important support for sustained antitumor immunity and, in some settings, may contribute substantially to tumor control. The study by Cho and colleagues provides compelling mechanistic evidence for the importance of CD4^+^ T cell help, showing that the inclusion of MHC-II-restricted neoantigens is critical for the activation of antigen-specific CD8^+^ T cells and for generating durable anti-tumor efficacy. The practical question for vaccine design is whether to emphasize MHC-I restricted epitopes, or MHC-II restricted epitopes, or both in roughly equal proportions.

A critical gap in the present evidence base is the predictive value of tumor-intrinsic biomarkers for vaccine response. Potential biomarkers of vaccine response include tumor mutational burden (TMB), microsatellite instability (MSI) status, PD-L1 expression and baseline immune infiltration pattern, but none of these has been prospectively validated in randomized trials of mRNA cancer vaccines4,83. The variation in clinical outcomes among tumor types, with melanoma and PDAC demonstrating the most promising results, while other tumor types have been more challenging, points to a role for tumor-intrinsic factors in vaccine efficacy. Identification of predictive biomarkers would enable enrichment of clinical trials in the patients most likely to benefit, and would facilitate selection of the most appropriate combination strategy for each patient.

The choice between self-amplifying mRNA (saRNA) and non-replicating mRNA platforms is a strategic decision with important implications for vaccine design and clinical development (109). Alphavirus genome-based saRNA constructs encode the viral replicase machinery as well as the antigen of choice, enabling intracellular amplification of the mRNA and much higher and sustained antigen expression from a lower initial dose. The possible advantages of saRNA include the ability to reduce the dose, prolong antigen expression and potentially allow for single-dose regimens. However, saRNA constructs are larger and more complex than non-replicating mRNA, pose additional challenges for manufacturing, and the replicase machinery itself may be immunogenic, which may limit the efficacy of repeated dosing. Clinical data comparing saRNA and non-replicating mRNA vaccines for cancer are limited, and the choice of platform is based on preclinical data and manufacturing considerations rather than proven clinical benefit.

The durability of vaccine-induced immune memory will be an important question answered by long-term follow-up of clinical trial participants. The existing clinical data, particularly from the phase I trial of autogene cevumeran in PDAC, indicate that neoantigen-specific T cells can persist for years following vaccination. However, the duration of these responses during continued antigen exposure from residual tumor cells, the impact of subsequent therapies on memory T cell populations, and the requirement for booster vaccinations to maintain protective immunity are all not well understood. Standardized immune monitoring assays applicable across multiple clinical trials would enable comparative analysis of the durability of immune memory across vaccine platforms and clinical settings.

The disparity between the preclinical efficacy and clinical translation is a recurrent theme in cancer vaccine research. In preclinical models, which frequently use implantable tumor cell lines in syngeneic mice, mRNA vaccination invariably leads to strong tumor rejection, with complete responses observed in most animals. However, the clinical responses in patients have generally been more modest than those observed in many preclinical models (110). Several reasons may explain this discrepancy: the relatively low mutational load of implantable murine tumors relative to human cancers, the lack of the chronic immune editing and immunosuppression that are typical of human tumors, the more homogeneous genetic background of inbred mouse strains, and the use of optimized vaccine dose schedules that may not be translatable to clinical practice. The advent of preclinical models that more closely reflect the complexity of human cancer, such as genetically engineered mouse models, patient-derived xenografts in humanized mice and ex vivo human tumor organoid-immune cell co-cultures, would enhance the predictive value of preclinical studies and decrease the attrition rate in clinical translation (28).

The evidence base is much stronger in some areas of mRNA cancer vaccine research than in others. The safety and immunogenicity of mRNA-LNP vaccines are well established. Billions of doses were administered during the COVID-19 pandemic and antigen-specific T cell responses are consistently observed in clinical trials. However, evidence for clinical efficacy is more limited, but is rapidly increasing with the results of the randomized phase II KEYNOTE-942 providing the strongest evidence to date. Preclinical models and early phase clinical trials provide the rationale for the relative efficacy of different LNP formulations, optimal neoantigen selection criteria and optimal combination strategies. However, data on the long-term durability of immune memory, the effect of mRNA vaccines on overall survival and the cost-effectiveness of personalized mRNA vaccines versus alternative therapies are still lacking and will require completion of ongoing phase III trials and long-term follow-up studies (111).

## Summary and future perspectives

Therapeutic mRNA vaccines for cancer have come to a pivotal inflection point. The convergence of developments in mRNA sequence engineering, LNP delivery technology, and immunomodulatory combination approaches has advanced this platform from a promising concept into a clinically tested therapeutic strategy. The randomized phase II data in melanoma, the demonstration of neoantigen-specific T cell responses and clinical benefit in pancreatic cancer, and the emerging evidence of efficacy in NSCLC and other solid tumors all establish that mRNA vaccines can generate clinically meaningful anti-tumor immune responses in patients. The platform-based manufacturing paradigm validated at an unprecedented scale during the COVID-19 pandemic provides the infrastructure for rapid, cost-effective production of personalized cancer vaccines.

The tripartite framework of antigen delivery and immunomodulation that has guided this review highlights priority areas for the next decade of research. In the antigen dimension, the most promising approach to overcome the antigenic heterogeneity that hampers the efficacy of single-target approaches is the development of multi-antigen, multi-target vaccines engaging both CD4^+^ and CD8^+^ T cell responses against a broad repertoire of tumor antigens, including TAAs, CTAs and personalized neoantigens. The combination of functional immunogenicity assays with neoantigen prediction pipelines and the use of machine learning algorithms based on clinical outcome data should improve the accuracy of neoantigen selection and enable the rational design of vaccine constructs with maximum immunogenic potential.

The next frontier in the delivery dimension is engineering of LNPs with tissue-specific and cell-type-specific tropism. The ability of the ionizable lipid linker chemistry to redirect LNP biodistribution from liver to lung provides proof of concept for the rational design of tissue-targeted delivery platforms. The development of LNPs that preferentially target DCs in lymphoid organs, together with optimization of the route of administration to maximize delivery to the lymphatic system, should improve the efficiency of T cell priming and reduce the required dose of mRNA for effective vaccination. The search for other ways of delivering drugs should continue, including biomimetic vectors, polymer-based systems and implantable devices that allow a sustained local release, with the aim of enlarging the delivery toolkit and allowing the choice of the optimal platform for each clinical indication.

A major focus of clinical research should be the systematic evaluation of combination strategies in the immunomodulation dimension, that is, coupling mRNA vaccines with immune checkpoint blockade, oncolytic virotherapy, innate immune agonists and adoptive cell therapy (89). The challenge is to find the best combination for each tumor type and clinical context, with the help of predictive biomarkers of response and resistance. Standardized immune monitoring assays applicable across multiple clinical trials should be developed to accelerate the discovery of immunomodulatory strategies most likely to benefit specific patient populations, along with systematic collection of tumor biopsy and blood samples for correlative biomarker analysis.

A revolutionary long-term goal is the development of prophylactic mRNA vaccines for individuals at high risk of cancer. The ability of mRNA vaccines to induce durable T-cell memory responses, together with the identification of shared neoantigens and cancer-predisposition mutations in high-risk populations (e.g., Lynch syndrome, BRCA1/2 mutations, and familial adenomatous polyposis), raises the possibility of intercepting cancer development at the premalignant stage through prophylactic vaccination. This approach could prevent cancer rather than treat established disease and therefore represents one of the most compelling long-term goals for the field, although significant biological, logistical, and ethical challenges remain to be overcome.

Advancing manufacturing technologies to point-of-care production is a key element for the broad clinical use of personalized mRNA vaccines. Shortening the manufacturing timeline from the current 4–6 weeks to 2–3 weeks, or even days, would substantially increase the clinical feasibility of personalized vaccination, especially for patients with rapidly progressive disease. This would require the establishment of automated, closed-system manufacturing platforms to be operated in hospital pharmacies, along with the validation of rapid-release analytical methods, to facilitate the decentralized production of personalized mRNA vaccines and alleviate the logistical burden of cold-chain distribution.

The next decade of mRNA cancer vaccine research should be aimed at systematic optimization of the tripartite design space by multi-antigen targeting, tissue-specific delivery engineering and rational combination immunotherapy. The ultimate goal is to translate therapeutic mRNA cancer vaccines from a promising investigational modality to a standard-of-care treatment that can be routinely offered to patients with cancer, and ultimately to extend the application of mRNA vaccine technology to the prevention of cancer in high-risk populations. The scientific, clinical and regulatory foundations for this transformation are now in place; the challenge for the field is to realize this vision with the rigor, collaboration and urgency that this promising technology deserves.
